# Removal of an Ovarian Tumor, Weighing 111 Pounds, from a Child

**Published:** 1893-08

**Authors:** W. W. Keen

**Affiliations:** Professor of the Principles of Surgery and of Chinical Surgery, Jefferson Medical College; Philadelphia


For Texas Medical Journal.

### AN OVARIAN TUMOR WEIGHING 111 POUNDS REMOVED FROM A CHILD OF 15, WHOSE WEIGHT WAS 68 POUNDS.

BY W. W. KEEN, M. D.,
Professor of the Principles of Surgery and of Chinical Surgery, Jefferson Medical College.

[Read before Academy of Surgery, Philadelphia.]

MISS B., of Benezette, Pa., was first seen by me at Driftwood, Pa., February 26, 1892, at the request of Dr. V. K. Corbett, of Caledonia. She was then fourteen years of age and had never menstruated. About eighteen months before I saw her, her abdomen began to enlarge. Six months afterward Dr. Corbett was consulted for an attack of considerable pain in the left side of the abdomen. He found that she was only voiding eight ounces of urine in twenty-four hours, but under proper treatment this soon reached a quart in amount, and has remained so ever since. He never discovered any albumin in the urine. In October, 1891, she had been tapped by a gynecologist, who is said to have diagnosticated a solid and probably malignant tumor, connected most likely with the liver, omentum, and ovary, and who deemed its removal not feasible.

I found the abdomen enormously distended with fluid, and advised very strongly that a small incision should be made in the abdominal wall, so that I could determine the relations of the growth with accuracy. Her father, however, was not present, and had made it a condition that nothing beyond tapping should be done. I tapped her immediately, and removed considerably over three gallons of amber-colored fluid. When this was evacuated, I discovered a lobulated tumor on the right side of the abdomen, under the liver and apparently attached to it. It was evidently cystic in part, there being at least two cysts perceptible. Each of these I tapped, obtaining from the upper one a light fluid, and from the lower one a much darker fluid. On account of her age no vaginal examination was made. The fluids pointed strongly toward an ovarian cystoma. I again advised an exploratory incision.

April 29, 1893. The patient was finally brought to the Jefferson College Hospital. She has been tapped twice since February, 1892, the last time in February, 1893, when six and a half gallons were drawn off. She is now enormously swollen. The measurements are as follows: From the ensiform to the umbilicus, 16½ inches; from the ensiform to the pubes, 29½ inches (this measurement in myself reaches from the ensiform to the middle of the calf of my leg); circumference, 49 inches. The veins over the abdomen are very large. Nothing can be made out in the interior in consequence of the enormous abdominal distension. Examination of the urine shows no albumin and a very slight trace of sugar. (?)

*Operation.* April 30, 1893. A small incision was made in the median line above the umbilicus, as the greater mass of the tumor lay there. A large trocar was thrust in and evacuated a very large quantity of characteristic opalescent ovarian fluid. The escape of this fluid revealed through the abdominal wall large masses lying especially under the liver and in the right iliac fossa. After this evacuation I enlarged the incision until it measured eventually about eight inches in length. I introduced my hand and found an enormous ovarian cyst, reaching up to the diaphragm and pushing everything out of its way. There were a number of moderate adhesions, chiefly to the belly wall and the omentum. The viscera were fortunately entirely free. The pedicle was only 2½ inches broad. The tumor arose in the right ovary, the left ovary being healthy but small.

The weight of the solid mass removed was twenty-seven pounds, and by actual weighing, the fluid removed weighed eighty-four pounds, making a total of 111 pounds. The child herself weighed but sixty-eight pounds.

After the removal of the tumor I never saw so curious a looking abdominal cavity. It looked almost like that of an eviscerated cadaver in the dissecting-room. The tumor had so pushed the liver to the right and backward, and the stomach to the left, that nearly the whole of the diaphragm was exposed, and flapped up and down with the pulsations of the heart. Down the middle of the cavity the bodies of the vertebra were entirely exposed, showing the aorta and vena cava to their bifurcations, the intestines being a very minor consideration, and pushed to each side in the hollow of the ribs and the lumbar region. When the abdominal wall was sutured the abdomen was excessively scaphoid, the anterior abdominal wall lying directly on the aorta and vertebræ. The puckering of the skin, although moderately marked, was much less than I had expected.

When the operation was completed, a glass drainage-tube was inserted, and she was put to bed in very fair condition, in view of the gravity of the operation. The tumor was a multilocular cyst.

May 18, 1893. The child has made an uninterrupted recovery. The drainage-tube was removed on the fifth day, when the discharge had become almost nothing, but three days later a slight rise of temperature took place, and the discharge recommenced. A small rubber drainage-tube was therefore reinserted for a few days. She sat up at the end of two weeks, and will go home as soon as the slight discharge from the drainage opening ceases.

REMARKS. I have not had time to search through the literature of ovariotomy, but so far as my memory serves I have never known of a larger tumor removed from a child. It weighed just one and a half times as much as the patient. Her recovery has been most satisfactory in spite of a very poor and capricious appetite. The chief lesson the case teaches is the value of an exploratory incision in every case of doubt. Had this been done, instead of a mere tapping, in October, 1891, when the tumor was much smaller, the prognosis would have been much more favorable, and she would have been spared a year and a half of needless suffering. What seemed to be a most formidable operation really proved to be almost simple one, the adhesions and the pedicle being most favorabe for the speedy recovery which has ensued.

